# Association between maternal age and outcomes in Kawasaki disease patients

**DOI:** 10.1186/s12969-019-0348-z

**Published:** 2019-07-19

**Authors:** Wei-Dong Huang, Yu-Ting Lin, Zi-Yu Tsai, Ling-Sai Chang, Shih-Feng Liu, Yi-Ju Lin, Ho-Chang Kuo

**Affiliations:** 1 Department of Pediatrics, Baoan Maternal and Child Health Hospital, Jinan University, Shenzhen, 518102 China; 2 Department of Medical Education, Kaohsiung Chang Gung Memorial Hospital, Kaohsiung, Taiwan; 3 Department of Pediatrics and Kawasaki Disease Center, Kaohsiung Chang Gung Memorial Hospital, and Chang Gung University College of Medicine, Kaohsiung, Taiwan; 4 Chang Gung University, College of Medicine, Taoyuan, Taiwan; 5 Department of Respiratory Therapy, Kaohsiung Chang Gung Memorial Hospital, Kaohsiung, Taiwan; 6 Division of Pulmonary & Critical Care Medicine, Department of Internal Medicine, Kaohsiung Chang Gung Memorial Hospital and Chang Gung University College of Medicine, Kaohsiung, Taiwan

**Keywords:** Kawasaki disease, Maternal age, Intravenous immunoglobulin, Coronary artery lesions

## Abstract

**Background:**

The etiology of Kawasaki disease (KD) is still unknown; perinatal factors may have role with few studies. This study was aim to survey the perinatal factors and clinical outcome of KD, including coronary artery lesion (CAL) formation and intravenous immunoglobulin (IVIG) treatment response.

**Methods:**

We enrolled a total of 185 KD patient–caregiver dyads in this study using questionnaires. The questionnaire included two categories: children’s characteristics, which consisted of age at disease onset, gender, gestational age at delivery, birth body weight, delivery methods, and breastfeeding status, and caregivers’ characteristics, which consisted of parents or not, education levels, maternal age at giving birth, total number of offspring, and family income. We analyzed the association of these factors with CAL formation and IVIG treatment response of KD.

**Results:**

KD patients with CAL formation had a higher maternal age than non-CAL patients (32.49 ± 3.42 vs. 31.01 ± 3.92 years, *p* = 0.016). We also found that maternal age ≥ 32 years group had a higher rate of having KD patients with CAL (39/81 vs. 24/74, odds ratio 1.935, 95% confidence interval [1.007, 3.718], *p* = 0.047). The maternal age ≥ 35 years group had a higher rate of having KD patients with IVIG resistance (6/31 vs. 6/116, odds ratio 4.400, 95% confidence interval [1.309, 14.786], *p* = 0.01). There was no significant difference in either CAL formation or IVIG resistance in KD with regard to patient’s age at disease onset, gestational age, birth body weight, delivery methods, breastfeeding, caregiver type, caregivers’ education level, total number of offspring, or family income (*p* > 0.05).

**Conclusions:**

This study is the first to report that maternal age is significantly associated with CAL formation and IVIG resistance in KD. We hypothesize that a maternal age less than 32 years would benefit KD offspring.

## Background

Kawasaki disease (KD) is a form of acute febrile systemic vasculitis that primarily affects children under the age of 5 years old [1]. The most serious sequela or complication to manifest is coronary artery lesions (CAL), including coronary artery dilatation, coronary artery aneurysm, and coronary artery fistula, all of which may have long-term consequences, such as stenosis or obstruction of coronary arteries and even myocardial infarction with time [2]. The efficacy of intravenous immunoglobulin (IVIG) administered to KD patients during the acute phase has been well established for reducing the prevalence of coronary artery abnormalities. However, IVIG resistance, the incidence of which varies widely among studies, occurs in approximately 9.4–38% of KD children [3, 4]. Despite decades of research, the etiology of KD remains unknown. The commonly accepted pathogenetic theory is currently that KD is a disease with autoimmune-autoinflammatory response that is triggered in genetically predisposed subjects by certain environmental factors [5, 6]. Nevertheless, exposure to no single environmental factor has yet been convincingly implicated in the etiology of KD. As indicated by Hayward et al. [7], since KD predominantly affects younger children, prenatal or perinatal factors may be relevant to its etiology. The role of perinatal exposure in offspring KD was evaluated in a study [7], with evidence showing a relationship between increased maternal age and subsequent KD. However, there were few data about the influence of perinatal exposure factors on CAL formation or IVIG response in KD.

In the past three decades, the mean maternal age of giving birth is increasing in many countries, especially in developed countries and Taiwan [8]. In particular, various studies have reported that advanced maternal age (defined as 35 years old or more) is associated with many adverse pregnancy outcomes [8]. Taking together, perinatal factors may influence health outcomes in offspring. Identifying the groups vulnerable to developing coronary artery lesions and having IVIG resistance in KD is important and more precise individualized medicine could be provided. Based on the hypothesis that perinatal factors and socioeconomic characteristics may influence the risk of subsequent KD by affecting the conditions of predisposition and immunity in offspring, this study aimed to survey the characteristics of both KD patients and their caregivers in KD disease outcomes.

## Methods

### Study design

We performed a retrospective cohort study at Kaohsiung Chang Gung Memorial Hospital in Taiwan that included pediatric KD patients/caregivers, with the KD patients having been diagnosed and followed between January 2014 and December 2017. The study consisted of patients diagnosed using the diagnostic criteria for KD established by the American Heart Association, with an International Classification of Diseases, 9th Revision, Clinical Modification, ICD-9-CM: 446.1 for KD. The study was approved by the Institutional Review Board of Chang Gung Memorial Hospital (IRB No.104-8261C), and we obtained written informed consent from all participants’ parents or legal guardians. The KD patients’ caregivers questionnaire were completed during study period and consisted of two categories: (1) children’s characteristics, which included age at disease onset (years), gender, gestational age at delivery, birth body weight, delivery methods (vaginal/cesarean), and breastfeeding status (yes/no) (duration) and (2) caregivers’ characteristics, which included caregiver (parents/non-parents), education levels (senior high school/university), maternal age of giving birth to the KD patient, total number of children born to each patient’s mother, and family income.

Both KD patients and their caregivers were grouped according to differences in CAL formation and IVIG treatment response. In this study, two-dimensional echocardiographic examinations were performed for all KD patients. CAL was defined as a lumen diameter greater than 3 mm in a child < 5 years of age or greater than 4 mm in a child ≥5 years of age, if the internal diameter of a segment was 1.5 times larger than that of an adjacent segment, or if the morphology of the coronary lumen was obviously irregular [2].

All patients were initially treated with a single infusion of IVIG (2 g/kg) over 12 h. Aspirin (3–5 mg/ kg / day) was administered after discharge until all signs of inflammation were resolved or CAL regression was detected on echocardiography. We recorded the primary clinical features and medication treatment response as well as disease outcome. IVIG-responsive KD was defined as patients who responded to a single dose of IVIG therapy, as shown by having no fever 48 h after IVIG and no recurrence of fever (> 38 °C) for at least 7 days after IVIG, as well as the apparent improvement or normalization of such inflammation signs as non-exudative conjunctival injection, cracked lips, strawberry tongue, erythema/induration at the bacillus Calmette Guérin inoculation site, polymorphous exanthema, and swollen hands and feet. IVIG-resistant KD was defined as patients who still require additional treatments after the initial IVIG therapy.

### Statistical analyses

All statistical analysis of the questionnaire outcomes was performed using SPSS software, version 17.0 (SPSS Inc., Chicago, IL, USA). We adopted the independent t-test and chi-square test (containing odds ratio (OR) and 95% confidence interval (CI)) in the characteristic profiles of the KD patients and their caregivers with regard to differences in CAL formation and IVIG treatment response. We assigned maternal age the cut-off value of 35 years based on the traditionally defined references of advanced maternal age as women aged 35 years or older at delivery [9]. A *P*-value of < 0.05 was considered statistically significant. The power of this study in CAL was 0.659 (alpha = 0.05, t-test).

## Results

A total of 185 KD patient–caregiver dyads were enrolled in this study. The characteristics of KD patients and their caregivers between the group with CAL and that without CAL are shown in Table 1. Male KD patients had a higher incidence of CAL formation than female ones (56/112 vs. 19/73, *p* = 0.001). KD with CAL formation also had a higher maternal age than non-CAL patients (32.49 ± 3.42 vs. 31.01 ± 3.92 years, *p* = 0.016). Furthermore, we found that the maternal age ≥ 32 years group had a higher rate of having KD patients with CAL (39/81 vs. 24/74, OR 1.935, 95% CI [1.007, 3.718], *p* = 0.047) when compared with the maternal age < 32 years group. There was no significant difference in CAL formation between KD patients with a maternal age ≥ 35 years and those with a maternal age < 35 years (*p* > 0.05).

**Table 1:** Children’s and caregivers’ characteristics among KD children with and without CAL

	With CAL (*n* = 75)	Without CAL (*n* = 110)	*P* value
Children’s characteristics			
Age, mean, years	1.83 ± 1.42	1.82 ± 1.55	.947
Gender, No.			.001
Male	56	56	
Female	19	54	
Gestational age, mean, weeks	38.37 ± 1.82	38.37 ± 2.18	.812
Prematurity, No.			.339
<37 weeks	6	14	
≧37 weeks	54	77	
Birth body weight, mean, grams	3128.66 ± 390.41	3023.76 ± 474.02	.146
Birth body weight, No.			.331
1500 g~ 2499 g or ≧4001 g	4	10	
2500 g~ 4000 g	60	83	
Delivery methods, No.			.163
Vaginal	43	52	
Cesarean	20	39	
Breast-feeding, mean, weeks	28.61 ± 42.88	29.04 ± 32.30	.951
Breast-feeding, No.			.776
Yes	51	70	
No	13	20	
Caregivers’ characteristics			
Caregiver, No.			.885
Parents	65	94	
Non-parents	6	8	
Education Levels, No.			.390
Senior high school	33	54	
University	42	53	
Maternal age, mean, years	32.49 ± 3.42	31.01 ± 3.92	.016^*^
Maternal age, No.			.047^*^
<32 years	24	50	
≧32 years	39	42	
Total number of children, mean	1.79 ± 0.66	1.90 ± 0.60	.236
Total number of children, No.			.827
≦2	65	96	
≧3	9	12	
Family income, No.			.450
≦49999 New Taiwan dollar per month	41	54	
≧50000 New Taiwan dollar per month	23	39	

Data are expressed as mean ± SD or n

Statistical values are expressed as t-value

*p < 0.05

The characteristics of KD patients and their caregivers between IVIG-responsive and IVIG-resistant groups are shown in Table 2. Compared with a maternal age less than 35 years old, the maternal age ≥ 35 years group had a higher rate of having KD patients with IVIG resistance (6/31 vs. 6/116, OR 4.400, 95% CI [1.309, 14.786], *p* = 0.01). There was no significant difference in the mean maternal age (31.7 years old) or in the cut-off (32 years old) maternal age between the IVIG responsive and resistant groups (*p* > 0.05).

**Table 2:** Children’s and caregivers’ characteristics among KD children with IVIG responsiveness and resistance

	IVIG responsiveness (*n* = 162)	IVIG resistance (*n* = 14)	*P* value
Children’s characteristics			
Age, mean, years	1.82 ± 1.51	1.89 ± 1.27	.88
Gender, No.			.133
Male	94	11	
Female	68	3	
Gestational age, mean, weeks	38.38 ± 2.01	38.38 ± 1.12	1.00
Prematurity, No.			.577
<37 weeks	17	1	
≧37 weeks	113	12	
Birth body weight, mean, grams	3080.44 ± 475.23	3033.69 ± 225.06	.727
Birth body weight, No.			.224
1500 g~ 2499 g or ≧4001 g	14	0	
2500 g~ 4000 g	122	13	
Delivery methods, No.			.405
Vaginal	81	8	
Cesarean	54	3	
Breast-feeding, mean, weeks	27.92 ± 34.77	52 ± 66.53	.101
Breast-feeding, No.			.059
Yes	109	7	
No	25	5	
Caregivers’ characteristics			
Caregiver, No.			.974
Parents	139	12	
Non-parents	12	1	
Education Levels, No.			.839
Senior high school	75	7	
University	84	7	
Maternal age, mean, years	31.39 ± 3.83	33.17 ± 4.17	.129
Maternal age, No.			.01^*^
<35 years	110	6	
≧35 years	25	6	
Total number of children, mean	1.88 ± 0.63	1.64 ± 0.63	.189
Total number of children, No.			.550
≦2	139	13	
≧3	20	1	
Family income, No.			.09
≦49999 New Taiwan dollar per month	80	10	
≧50000 New Taiwan dollar per month	57	2	

Data are expressed as mean ± SD or n

Statistical values are expressed as t-value

*p < 0.05

The main results of the group with a cut-off maternal age of 32 years and that of 35 years were independent of the potential confounders evaluated. The associations showed no significant difference in different maternal age groups (32 or 35 years) regarding patient’s age at disease onset, gestational age, birth body weight, delivery methods, breastfeeding, total number of offspring born to each patient’s mother, or family income (*p* > 0.05).

There was no significant difference in either CAL formation or IVIG resistance in KD related to age at disease onset, gestational age, birth body weight, delivery method, or breastfeeding. As for caregivers’ characteristics, we observed no significant difference in either CAL formation or IVIG resistance in KD with regard to caregiver type (parents/non-parents), caregivers’ education level, total number of offspring born to each patient’s mother, or family income.

## Discussion

In this study, we demonstrated relationships between clinical outcomes of KD (including CAL formation and IVIG treatment response) and various potential perinatal and socioeconomic characteristics of both KD patients and their caregivers. We found that increasing maternal age was associated with both CAL formation and IVIG resistance in KD patients but observed different cut-off maternal ages. Using the traditional cut-off value of advanced maternal age (≥ 35 years), our results showed that mothers aged 35 years or older had a higher rate of having KD patients with IVIG resistance when compared to a maternal age less than 35 years. We also found a significant difference in the CAL formation of KD when the cut-off value of maternal age was set as 32 years, which was also the median maternal age in this study. To the best of our knowledge, the observed cut-off maternal age difference between CAL formation and IVIG resistance is a novel finding that could not be explained by such confounding factors as each patient’s age at disease onset, gestational age, birth body weight, delivery methods, breastfeeding, total number of offspring born to each patient’s mother, or family income. Hayward et al. [7] collected perinatal data that reported the influence of maternal age in KD and evidence of increasing maternal age and KD in offspring, especially at the maternal age of 35 years or more [7]. However, no analyzed data was available for the status of either CAL or IVIG response in KD patients with maternal age.

KD is reported to be a disease with autoimmune-like or autoinflammatory response that is triggered in genetically predisposed subjects by certain environmental factors [5, 6]. Growing evidence has shown a link between KD and immune-mediated diseases, especially allergic diseases or autoimmune diseases. Children with allergic diseases like urticaria, allergic rhinitis, and atopic dermatitis are subsequently at an increased risk of KD [10]. In contrast, a subsequent risk for allergic diseases including asthma [11], allergic rhinitis [11], and atopic dermatitis [12] has been found to be increased in patients with a history of KD. As for immune-mediated diseases, previous studies have indicated associations with increasing maternal age at delivery with an increased risk for type 1 diabetes [13] and food allergies [14] in the offspring. A fine-tuned balance between genetic, immunological, metabolic, and hormonal factors is necessary for reproduction, and all of these factors are likely involved in the aging process [15]. Therefore, maternal aging may affect health outcomes in offspring, including with regard to the development of their immune response. Furthermore, previous studies showed that increasing maternal age may be an indicator of accumulated exposure to environmental toxins or infections [16]. Another study speculated that factors related to older maternal age may affect the maturation of an offspring’s immune system [17]. Moreover, previous study by Burch et al. [18] showed that maternal age was associated with a decreased concentration of transforming growth factor-β1 in breast milk. Decrease in the serum levels of transforming growth factor-β1 in KD patients has also been reported [19]. Perhaps for the reason that maternal age plays a role in the concentration of inflammatory cytokines in breast milk, we find the association between maternal age and outcomes in KD patients. Based on the above, we suggested that increasing maternal age may potentially contribute to affecting the offspring’s immune system, possibly increasing their predisposition to both CAL formation and IVIG resistance in KD.

Genetically determined dysregulation of the immune system is an important factor in the pathogenesis of KD [5]. Many genetic association studies have been published with regard to both vasculopathy and IVIG response in KD patients. A study by Shrestha et al. [20] indicated that excessive transmission of *FCGR3B-NA1* was observed among KD patients with coronary artery disease and IVIG nonresponse. Onouchi et al. [21] reported that single-nucleotide polymorphisms in immune response genes ITPKC and CASP3 have been associated not only with an increased risk of CAL, but also with an increased risk of IVIG unresponsiveness in KD [21]. Many similar laboratory predictors of CAL formation and resistance to IVIG therapy in KD patients have also been identified. Higher C-reactive protein [3, 22, 23] and lower serum albumin [23, 24] have been proposed as having an association with both CAL formation and IVIG unresponsiveness in KD patients. High parameter of inflammation at diagnosis is one of the most important values for the purpose of decisions on the possible implementation of therapy in clinical practice, as C-reactive protein and erythrocyte sedimentation rate are the major factors suggested by American Heart Association for incomplete KD [25]. Clinical risk factors influencing CAL development and IVIG resistance have also been reported among KD patients. Long duration of fever has also been demonstrated as a risk factor for coronary involvement in many previous studies [26]. Furthermore, KD patients with persistent or recrudescent fever after a first course of IVIG therapy are well known to have a higher risk for developing CAL [27]. This association makes it appear reasonable that some genetic, laboratory and clinical predictors have similar tendencies in association with both CAL formation and IVIG resistance. Although the relation between these predictors and maternal age was still unknown, prenatal and perinatal factors could affect offspring autoimmune-autoinflammatory disorders. This was compatible with the current line of research on “the first 1000 days of life” as responsible of the global child health. Therefore, the advanced maternal age may be associated with poor outcomes in KD.

The protective roles of breastfeeding in the development of KD have been reported previously [28]. However, the effect of breastfeeding on the outcomes of KD may be different from that on the development of KD. Our data regarding breastfeeding showed that there was no significant difference between KD patients with and without CAL, as was found between KD patients with and without IVIG resistance. Meyer et al. [29] also reported no significant protective effect of breastfeeding on developing coronary artery aneurysm and being refractory to IVIG treatment. Moreover, according to the ‘hygiene hypothesis’, the microbes seeding the intestine during either cesarean delivery or vaginal delivery may alter long-term intestinal colonization and subsequently influence the postnatal development of immune system [30]. However, there was no data investigating the effects of delivery mode in KD in the literature. In this study, we didn’t find the protective role of vaginal delivery with significant better outcomes, though additional investigations would be necessary.

This study has certain limitations. First, sparse data for extreme maternal ages may lead to a sampling bias. This sampling bias may explain why our cut-off value was 32 years when we analyzed CAL formation, without a statistically significant difference with regard to the cut-off values of ages over 35 years. Second, parents might differ in their ability to remember or report depending on their age and socioeconomic status, which may lead to recall bias. Third, our data is also lacking information about race, birth order, some missing data and time to diagnosis.

## Conclusions

In conclusion, this study was the first to report that increasing maternal age has a significant association with CAL formation and IVIG resistance in KD. We hypothesized that maternal age under 32 years will benefit disease outcome in KD offspring. The exact mechanisms mediating the effect of increasing maternal age and the difference in the maternal age cut-off on the risk for CAL formation and IVIG resistance in subsequent KD observed in our study remains unclear. More studies with larger number of patients are needed to corroborate these findings. KD patients with advanced maternal age are in high risk in CAL formation and IVIG resistance; therefore, more aggressive therapies or examination arrangement may be needed for these patients.

## Data Availability

The datasets used and/or analyzed during the current study are available from the corresponding author on reasonable request.

## Abbreviations

CAL: Coronary artery lesions
IVIG: Intravenous immunoglobulin
KD: Kawasaki disease
